# The impact of spatial and verbal working memory load on semantic relatedness judgements

**DOI:** 10.3758/s13423-023-02323-0

**Published:** 2023-09-18

**Authors:** Dmytro Khanzhyn, Walter J. B. van Heuven, Karolina Rataj

**Affiliations:** 1 Faculty of English, Adam Mickiewicz University, Poznań, Poland; 2 School of Psychology, University of Nottingham, Nottingham, UK

**Keywords:** Semantic relatedness, Verbal working memory, Spatial working memory, Working memory load

## Abstract

Studies using a relatedness judgement task have found differences between prime-target word pairs that vary in the degree of semantic relatedness. However, the influence of working memory load on semantic processing in this task and the role of the type of working memory task have not yet been investigated. The present study therefore investigated for the first time the effect of working memory load (low vs. high) and working memory type (verbal vs. spatial) on semantic relatedness judgements. Semantically strongly related (e.g., *hip – KNEE*), weakly related (e.g., *muscle – KNEE*) and unrelated (e.g., *office – KNEE*) Polish word pairs were presented in an experiment involving a dual working memory and semantic relatedness task. The data revealed that, relative to semantically unrelated word pairs, responses were faster for strongly related pairs but slower for weakly related pairs. Importantly, the verbal working memory task decreased facilitation for strongly related pairs and increased inhibition for weakly related pairs relative to the spatial working memory task. Furthermore, working memory load impacted only weakly related pairs in the verbal but not in the spatial working memory task. These results show that working memory type and load influence semantic relatedness judgements, but the direction and size of the impact depend on the strength of semantic relations.

## Introduction

A well-established finding in the literature is that people are faster and more accurate at recognizing target words (e.g., *coffee*) that are preceded by semantically or associatively related primes (e.g., *tea*) relative to unrelated primes (e.g., *sky*) (for reviews, see McNamara, 2005; Neely, 1991). This phenomenon, referred to as semantic priming, has frequently been examined with the lexical decision task (LDT), in which participants decide whether or not a target word is a real word (e.g., Hutchison et al., 2013; Lucas, 2000; Neely, 1976). Another task that has often been used to investigate semantic processing and the effects of word relatedness is the semantic relatedness task (SRT), in which participants decide whether or not pairs of words are semantically related (e.g., Balota & Paul, 1996; Faust & Lavidor, 2003; Gilbert et al., 2018; Kuperberg et al., 2008; Ortu et al., 2013). One of the advantages of the SRT is that it more closely resembles natural language processing because participants need to explicitly access the meaning of both words in a pair to perform the task (Balota & Paul, 1996; Poort & Rodd, 2019).

Neuroimaging data presented in Kuperberg et al. (2008) revealed a characteristic pattern of brain activity involved in the SRT. Enhanced activation in the left parietal area in their study suggests that explicit relatedness judgements require more attention to semantic matching between primes and targets. Furthermore, the comparison of the reaction time data between the SRT and the LDT in Kuperberg et al.’s (2008) study with directly (e.g., *tiger – stripes*) and indirectly (e.g., *lion – stripes*) semantically related pairs revealed a larger priming effect for directly related pairs in SRT than in LDT and a reverse priming effect for indirectly related pairs in SRT, but no effect in LDT. These results suggest that the priming effect may be modulated by both task and the type of relatedness. The degree of associative prime-target relatedness was also shown to affect semantic processing in the SRT (Ortu et al., 2013). A graded effect of associative strength was found, with the largest facilitation effect observed for highly associated (e.g., *cherry – tree*) and a smaller effect for moderately associated (e.g., *camera – lens*) pairs as compared to unrelated ones. A different pattern of effects for indirectly related and moderately associated pairs in the SRT demonstrates that the intermediate relatedness condition may be particularly sensitive to task demands.

The differences in the processing of related and unrelated pairs have been explained through spreading activation in a network in which semantic information is stored in the form of interconnected nodes or concepts (Collins & Loftus, 1975). When a concept is activated, its activation spreads to related concepts, which become pre-activated and can be processed faster. There is evidence from the literature that the spread of semantic activation can be influenced by secondary tasks and strategic processes (for a review, see McNamara, 2005). For example, semantic priming effects can be modulated, at least partly, by high-order executive functions, such as executive control (Radel et al., 2015), attentional control (Hutchison et al., 2014) and working memory (Heyman et al., 2015).

To our knowledge, no experimental studies have so far investigated the influence of executive functions on semantic relatedness judgements. Studies on the impact of working memory (WM) load on semantic processing have revealed mixed outcomes. For example, Heyman et al.’s (2015) study was conducted with native Dutch speakers who completed five successive lexical decision trials after memorizing an easy (low-load condition) or complex (high-load condition) dot pattern. The materials included prime-target pairs with forward (FA; e.g., *panda – bear*), backward (BA; e.g., *ball – catch*) and symmetrical (SYM; e.g., *answer – question*) association. Consistent with Hutchison et al. (2014), who found a correlation between the level of attentional control and semantic priming, Heyman et al.’s (2015) results revealed that priming effects were almost eliminated for FA pairs in the high-load condition compared to the low-load condition, whereas priming effects were the same for BA and SYM pairs in both load conditions.

Heyman et al. (2017) conducted two further semantic priming experiments aiming to replicate Heyman et al.’s (2015) findings. Both experiments involved the same design and procedure as the original study. The first experiment was conducted in English, whereas the second experiment was a very precise replication of the original study in Dutch, but with a larger sample. Despite the similarities, Heyman et al. (2017) failed to replicate Heyman et al.’s (2015) crucial three-way interaction between load, relatedness and the type of association. As acknowledged by the authors, a possible reason for the unstable findings may be the non-verbal nature of the dot memory task used to manipulate WM load in both studies. It is also possible that the SRT may be a more suitable task for capturing the effects of additional WM load on semantic processing because it requires participants to deliberately process and remember both words in a pair.

Some previous studies used the SRT to explore semantic processing in word pairs that were directly or indirectly semantically related (Kuperberg et al., 2008) or that differed in association strength (Ortu et el., 2013), but they did not investigate the impact of WM. Studies that focused on the effect of WM on semantic processing did not distinguish between spatial and verbal domains and used only related and unrelated word pairs (Heyman et al., 2015, 2017). To explore the impact of verbal and non-verbal WM tasks on relatedness judgements, the present study manipulated WM type (spatial vs. verbal) and WM load (low vs. high) in an SRT with strongly related (SR), weakly related (WR) and unrelated (UR) pairs. To ensure consistent WM load across trials, participants performed a dual task in which the SRT was interleaved with a spatial or verbal WM task in each trial.

An overall effect of WM load on relatedness judgements was expected, i.e., participants were expected to be slower and less accurate in their responses when their WM was taxed more. Furthermore, we expected that the verbal WM task would have a larger impact on relatedness judgements than the spatial WM task because it additionally constrains resources in the verbal domain that are relevant for semantic processing. WR pairs were expected to be more sensitive to the WM manipulation due to weaker semantic links between the words. If WM load significantly impacts relatedness judgements, it will support the assumption that the spread of semantic activation can be affected by high-order executive functions (Heyman et al., 2015; Hutchison, et al., 2014; Radel et al., 2015).

## Methods

### Participants

Sixty-six students of the Faculty of English at Adam Mickiewicz University in Poznan who were native Polish speakers (58 women, one preferred not to state, *M*_*age*_ = 21.2 years, *SD*_*age*_ = 1.3 years) took part in the experiment. Data from four participants who gave less than 80% of correct responses to UR and SR pairs were excluded from analysis, which resulted in a final sample of 62 participants. They received course credits in return for their participation.

### Design

The experiment was designed using PsychoPy (Peirce et al., 2019) and conducted online on pavlovia.org. It consisted of two sessions separated by at least 7 days (maximum 23 days). Participants performed an SRT combined with a spatial WM task in one session and with a verbal WM task in the other session. Each session was divided into two blocks, each having a different level of WM load (low vs. high). The load was manipulated using the n-back paradigm (Kane et al., 2007). In the low-load block, participants identified the item (dot position or letter identity) from the previous trial (1-back). In the high-load block, participants identified the item from two trials before (2-back). The order of sessions and blocks within each session was counterbalanced across participants.

### Materials

Stimulus materials (word pairs) for the SRT were taken from Rataj et al. (2023). These word pairs (prime-target) were developed using semantic vectors and tested in two rating studies that differed only in the order in which the two words were presented (target-prime in Survey 1 and prime-target in Survey 2). Although semantic vectors are direction-agnostic, the ratings showed symmetric relationships. Critical Polish stimuli included 216 triplets consisting of a target word preceded by an SR word (e.g., *hip – KNEE*), a WR word (e.g., *muscle – KNEE*), and a UR word (e.g., *office – KNEE*). A further 16 unrelated word pairs were used as filler items that were presented after self-paced breaks in each block. Characteristics of the stimuli are presented in Table 5 in Appendix A. The stimuli were divided into eight lists, each containing 27 SR, 27 WR, and 54 UR pairs. Lists were assigned to participants so that targets did not repeat across blocks within each session. However, targets were repeated in the second session at least 7 days after the first session, but targets were preceded by a different word in the second session. This design ensured more than 1,600 observations for the critical WR and SR conditions in each working memory condition (27 × 62 = 1,674), which according to Brysbaert and Stevens (2018) is the recommended minimum for repeated-measures studies aiming to detect small effect sizes (*d* = .1) usually observed in reaction time experiments.

The stimuli for the spatial WM task were black dots located in one of eight pre-defined positions on the screen that formed a virtual circle. This layout was to ensure that participants did not engage in a verbal activity by verbalizing the locations. The stimuli for the verbal WM task included eight phonologically distinct letters presented one per trial at the centre of the screen. The letter position did not overlap with the prime/target position. The location of the letters was fixed across trials to prevent participants from using spatial WM.

The order of the stimuli in the SRT and WM tasks was pseudorandomized using the pseudorandom list generator (van Heuven, 2020). In the WM tasks, the number of “same” or “different” responses was equal. No more than four word pairs with the same degree of relatedness were presented in a row in the semantic relatedness task.

### Procedure

Each trial started with an SRT in which participants were shown a fixation cross, followed by a blank screen. Next, words in each word pair were presented one at a time. The first word of the word pair was presented in lowercase, followed by a blank screen and then the next word of the word pair (target) was presented in uppercase. Participants decided whether the target was semantically related to the lowercase word presented before the target by pressing ‘P’ for related or ‘Q’ for unrelated. After the participants’ response, or 3,000 ms, the verbal or the spatial WM task was presented depending on the session.

**Fig. 1:**
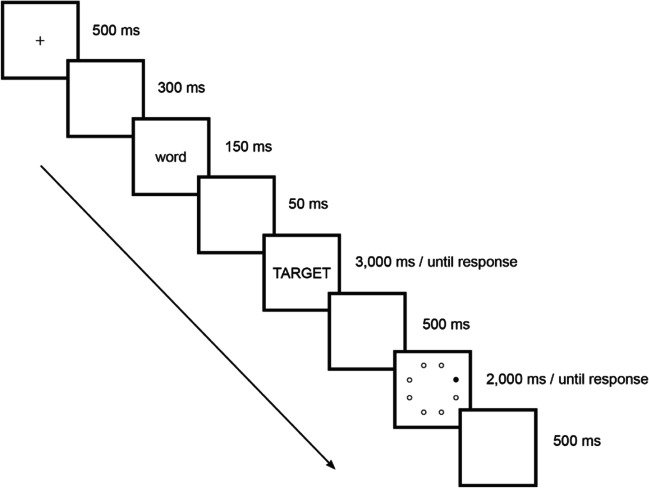
Experimental procedure of a spatial working memory trial

**Fig. 2:**
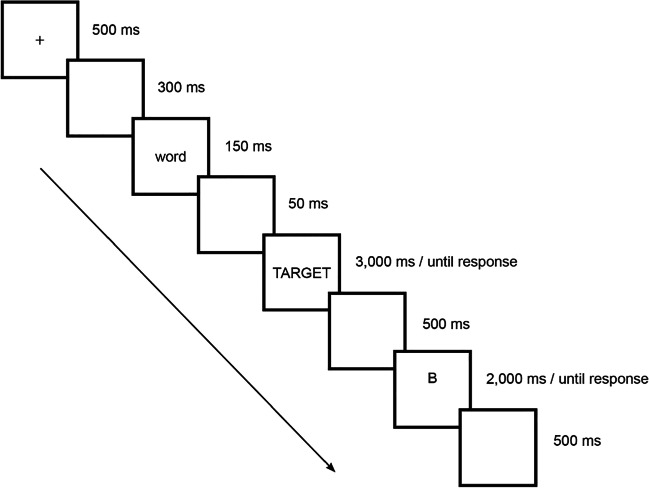
Experimental procedure of a verbal working memory trial

In the spatial WM task, participants decided whether the dot that they currently saw was in the same or a different position compared to the previous trial (low-load condition) or two trials before (high-load condition). The procedure for a spatial WM trial is illustrated in Fig. 1. In the verbal WM task, participants decided whether the letter that they currently saw was the same or different compared to the previous trial (low-load condition) or two trials before (high-load condition). The procedure for a spatial WM trial is illustrated in Fig. 2. Participants were instructed to press ‘P’ for same position/letter and ‘Q’ for different position/letter. The items remained on the screen until a response was made, but for no longer than 2,000 ms.

At the beginning of each block, participants completed a practice session to get familiar with the dual task receiving feedback on whether their answer was correct, incorrect, or too slow. Eighty percent of correct responses were required to proceed to the experimental session.

## Results

The reaction time (RT) analysis was conducted after removing incorrect responses and outliers. Outliers were removed based on a cut-off value of 2 standard deviations from the mean for each participant and target word in each condition. In addition, fast responses (< 250 ms) were removed. In total, 1.37% of responses as well as fillers were excluded from the analysis.

The data were analysed using generalised linear mixed-effects models (GLMMs) in R version 4.1.1 (R Core Team, 2021) with the lme4 package version 1.1.27.1 (Bates et al., 2015). GLMMs were used because they do not require the assumption of normal RT distribution, thus making it possible to analyse untransformed RT data (Lo & Andrews, 2015; Lupker et al., 2020).

### Working Memory (WM) tasks

Fixed effects in the models for RT and error analysis included Load (low vs. high), Task (spatial vs. verbal) and their interaction. The factors were coded using sum coding (-.5 vs. .5). Because models with random slopes failed to converge, the final models included only random intercepts for subjects and items. The model for the error analysis failed to converge with the default optimizer, so the BOBYQA optimizer was used. Mean RTs and error rates are presented in Table 1.

**Table 1:** Mean response times (RTs; in ms) and error rates (in %) in the working memory task as a function of task and load. Values in parentheses indicate standard errors

		Spatial task	Verbal task
Low load	RT Error rate	676 (2.7) 4.3 (0.26)	692 (2.57) 4.7 (0.28)
High load	RT Error rate	839 (2.73) 10.5 (0.4)	898 (2.98) 14.3 (0.47)

Participants were significantly slower (*b* = 184.37, *SE* = 1.54, *t* = 119.43, *p* < 0.001) and made more errors (*b* = -1.1, *SE* = 0.05, *z* = -20.79, *p* < 0.001) in the 2-back task as compared to the 1-back version regardless of the type of WM involved. Responses in the verbal task were slower (*b* = 37.18, *SE* = 1.48, *t* = 25.19, *p* < 0.001) and error rates were significantly higher (*b* = -0.12, *SE* = 0.03, *z* = -4.35, *p* < 0.001) than in the spatial task. The interaction between Task and Load was also significant for both RTs (*b* = 43.11, *SE* = 1.66, *t* = 26.02, *p* < 0.001) and error rates (*b* = -0.13, *SE* = 0.05, *z* = -2.42, *p* = 0.02). Bonferroni-corrected pairwise comparisons revealed a significant difference between verbal and spatial tasks in both load conditions in terms of RTs (16 ms, *p* < .001 for low load vs. 59 ms, *p* < .001 for high load) and only in the high-load condition in terms of error rates (0.4%, *p* = 0.25 for low load vs. 3.8%, *p* < .001 for high load).

### Semantic relatedness task

#### Response Time (RT) analysis

Fixed effects in the model included Load (low vs. high), Task (spatial vs. verbal), Relatedness (UR vs. SR vs. WR) and their interactions. The factors Load and Task were coded using sum coding (-.5 vs. .5), whereas Relatedness was coded using deviation coding with the unrelated condition as the reference. The BOBYQA optimizer was used because the model with the default optimizer failed to converge. RT means and standard errors are summarised in Table 2 and the final model output is presented in Table 3.

**Table 2:** Mean response times (RTs; in ms) and error rates (in %) in the semantic relatedness task as a function of working memory task, working memory load and prime-target relatedness. Values in parentheses indicate standard errors

		Spatial task		Verbal task	
		Low load	High load	Low load	High load
SR	RT Error rate	813 (3.76) 7.3 (0.64)	885 (4.77) 7.2 (0.64)	834 (3.83) 8.1 (0.67)	913 (5.08) 9.5 (0.73)
WR	RT Error rate	919 (3.05) 28.4 (1.11)	989 (3.08) 30.6 (1.13)	930 (3.41) 29.3 (1.12)	1036 (3.64) 32.6 (1.15)
UR	RT Error rate	870 (3.97) 0.6 (0.13)	938 (2.93) 2.1 (0.25)	867 (3.42) 1.0 (0.18)	944 (3.03) 4.4 (0.36)

*SR* strongly related pairs, *WR* weakly related pairs, *UR* unrelated pairs

**Table 3:** Generalised linear mixed-effects model (GLMM) output for the response time (RT) analysis in the semantic relatedness task

Factor	*b*-value	SE	*t*-value	*p*-value
Task	18.505	1.708	10.833	< 0.001
Load	79.012	2.244	35.204	< 0.001
Relatedness (WR vs. UR)	63.574	2.089	30.439	< 0.001
Relatedness (SR vs. UR)	-43.748	2.552	-17.142	< 0.001
Task × Load	17.783	2.271	7.829	< 0.001
Task × Relatedness (WR vs. UR)	27.766	2.487	11.163	< 0.001
Task × Relatedness (SR vs. UR)	23.4	2.451	9.547	< 0.001
Load × Relatedness (WR vs. UR)	14.528	2.047	7.096	< 0.001
Load × Relatedness (SR vs. UR)	2.835	3.668	0.773	0.44
Task × Load × Relatedness (WR vs. UR)	27.6	1.917	14.398	< 0.001
Task × Load × Relatedness (SR vs. UR)	-1.206	2.198	-0.549	0.58

*SR* strongly related pairs, *WR* weakly related pairs, *UR* unrelated pairs

Final model: glmer(rt ~ task * load * relatedness + (1|subject) + (1|item), data = SRT.data, family = Gamma(link="identity"), control = glmerControl(optimizer = "bobyqa", optCtrl = list(maxfun=1e6))).

**Fig. 3:**
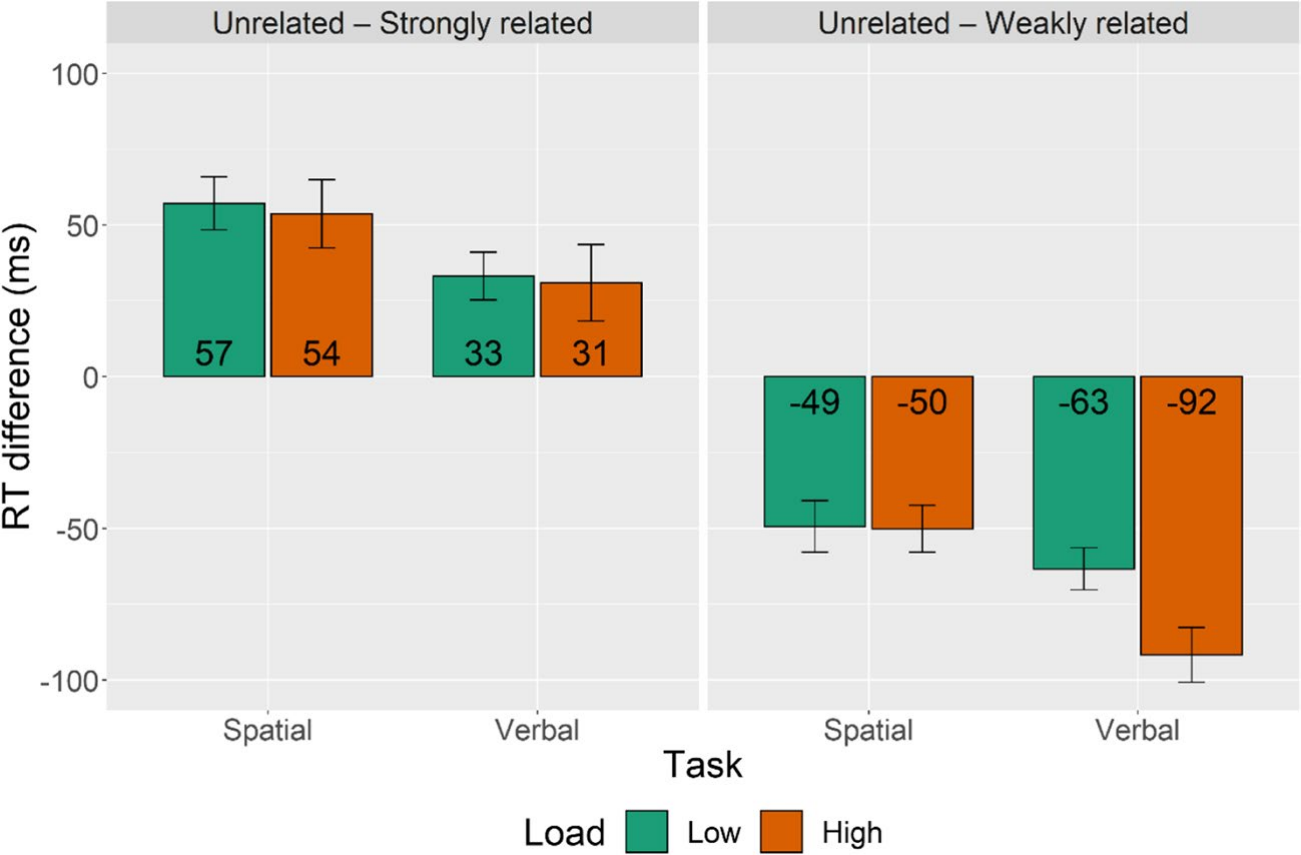
Response time (RT) differences for semantically strongly and weakly related pairs relative to unrelated pairs as a function of type of working memory (WM) task and load. Positive values indicate facilitation, whereas negative values indicate inhibition relative to unrelated pairs. Error bars indicate 95% confidence intervals

The analysis revealed significant main effects of Load, Task and Relatedness (*p*s < 0.001). There was an interaction between Load and Task because the effect of load was larger in the verbal than spatial WM task (88 ms vs. 70 ms, *p* < 0.001). Furthermore, there were interactions between Task and Relatedness for SR versus UR pairs because the facilitation effect was smaller with the verbal than with the spatial WM task (32 ms vs. 55 ms, *p* < 0.001), and for WR versus UR pairs because the inhibition effect was larger with the verbal than with the spatial WM task (-78 ms vs. -50 ms, *p* < 0.001). The interaction between Relatedness and Load was only significant for WR pairs and revealed that the inhibition effect was larger with the high-load than low-load WM task (-71 ms vs. -56 ms, *p* < 0.001). Crucially, there was a three-way interaction between Task, Load and Relatedness for WR pairs (*t* = 14.4, *p* < 0.001) but not for SR pairs (*t* = -0.55, *p* = 0.58). As can be seen in Fig. 3, for WR pairs there was no impact of load with the spatial WM task (-50 ms vs. -49 ms, *p* = 0.76), whereas with the verbal task a high memory load resulted in a stronger inhibition effect than a low memory load (-92 ms vs. -63 ms, *p* < .001).

#### Error analysis

Mean error rates are presented in Table 2 and the output of the GLMM analysis is presented in Table 4. The model included the same fixed and random effects as the model for the RT analysis. The BOBYQA optimizer was used, and the binomial family was used for the accuracy data.

**Table 4:** Generalised linear mixed-effects model **(**GLMM) output for the error analysis in the semantic relatedness task

Factor	*b*-value	SE	*z*-value	*p*-value
Task	-0.32680	0.06538	-4.998	< 0.001
Load	-0.55838	0.06539	-8.539	< 0.001
Relatedness (WR vs. UR)	-3.54600	0.08743	-40.558	< 0.001
Relatedness (SR vs. UR)	-1.77781	0.09397	-18.919	< 0.001
Task × Load	-0.12444	0.13077	-0.952	0.34
Task × Relatedness (WR vs. UR)	0.67013	0.17285	3.877	< 0.001
Task × Relatedness (SR vs. UR)	0.52702	0.18801	2.803	< 0.01
Load × Relatedness (WR vs. UR)	1.29235	0.17257	7.489	< 0.001
Load × Relatedness (SR vs. UR)	1.35852	0.18772	7.237	< 0.001
Task × Load × Relatedness (WR vs. UR)	0.06880	0.34501	0.199	0.84
Task × Load × Relatedness (SR vs. UR)	-0.07055	0.37520	-0.188	0.85

*SR* strongly related pairs, *WR* weakly related pairs, *UR* unrelated pairs

There were main effects of Load and Task (*p*s < 0.001) with more errors observed when SRT was interleaved with the 2-back WM task relative to the 1-back one and with the verbal WM task relative to the spatial one. More errors were found for WR and SR pairs relative to UR (*p*s < 0.001). The interactions between Load and Relatedness and between Task and Relatedness were significant for both WR pairs (*p*s < 0.001) and SR pairs (*p* < 0.001 and *p* < 0.01, respectively). The interaction between Task and Load and the three-way interaction between Task, Load and Relatedness were not significant (*p*s > 0.3).

## Discussion

The aim of this study was to investigate the effect of spatial and verbal WM load on semantic relatedness judgements with semantically SR and WR word pairs. The results revealed that the type of WM task impacts relatedness judgements of SR and WR pairs differently. With the verbal WM task, the facilitation effect for SR pairs was weaker than with the spatial WM task, whereas the inhibition effect for WR pairs was larger with the verbal WM task than with the spatial WM task.

The verbal task involved remembering letters and thus imposed additional constraints on resources required for semantic processing. The results suggest that semantic links between related words are particularly modulated by WM demands in the verbal domain. This may be because activation spreading between prime and target or the activation of the target becomes impeded when a WM task involves shared verbal resources. It is therefore possible that the type of WM taxed in the concurrent task rather than the difficulty of the task is important for semantic processing.

The type of task used in our study may also have contributed to the effects of additional spatial and verbal WM load on semantic processing. The nature of the SRT requires participants to remember the first word and process the meaning of two words to make a decision about the relatedness of the word pair. This decision is different from the one in the LDT, in which it is the lexical status of the target item that is evaluated. The WM demands inherent in relatedness judgements appear to make semantic processing in the SRT more sensitive to concurrent WM load. Whereas the LDT is appropriate for exploring the differences between automatic and strategic processes involved in semantic priming (Heyman et al., 2015, 2017), the SRT is likely more suitable for studying possible effects of different types of WM load on semantic processing.

Importantly, whereas the WM task had a significant effect on both relatedness conditions, a three-way Load × Task × Relatedness interaction was significant for WR but not for SR word pairs. The more difficult 2-back WM task increased the inhibition effect in the verbal but not in the spatial condition (see Fig. 3). This effect could be explained by the weaker semantic links between primes and targets in WR pairs that become more difficult to judge under increased WM load in the shared verbal domain.

As expected, participants were significantly faster in their responses to SR than to UR pairs. Interestingly, however, responses to WR pairs were overall slower than to UR pairs regardless of the WM type and load. To our knowledge, only two previous studies used an SRT with WR pairs. A linear priming pattern was reported by Ortu et al. (2013) with the fastest responses to SR pairs, slower responses to WR pairs and the slowest responses to UR pairs. In contrast, Kuperberg et al. (2008) found an inhibition effect for WR pairs. The materials and the procedure of our study more closely resemble Kuperberg et al. (2008) because participants immediately responded to target words, each of which was preceded by semantically strongly and weakly related primes. These similarities may explain a comparable pattern of results for WR pairs.

Overall, participants in our experiment found it difficult to decide whether WR pairs were related because the average error rate was 30%. It might be more appropriate to treat the accuracy rate for WR pairs in the SRT as a measure of consistency rather than correctness of responses. Kuperberg et al. (2008) also underlined the subjectivity of responses to the indirectly related condition and reported an even higher error rate of 50% for indirectly related pairs in the SRT.

A possible explanation for the larger percentage of errors and the inhibition effect for WR pairs is that the first word in these pairs (prime) may pre-activate words with stronger semantic links than the actual target word. When the target word is presented, the pre-activation of more strongly related concepts needs to be suppressed before making a decision about the weakly related target. The judgements of WR pairs are also particularly modulated by the concurrent high verbal WM load. Alternatively, when presented with a weakly related target word, participants may be searching for mediating concepts between the prime and the target, which may also require additional WM resources.^1^

Overall, the present findings provide new evidence about the influence of verbal WM load on semantic relatedness judgements and, more generally, support the view that semantic processing can be modulated by high-level cognitive functions (Heyman et al., 2015; Hutchison et al., 2014; Radel et al., 2015). Our results are also consistent with the assumption of the functional separability of spatial and verbal working memory resources (Baddeley et al., 2020; Shah & Miyake, 1996) and demonstrate that domain-specific working memory load may impact semantic processing.

## Conclusions

The present study revealed that semantic processing is influenced more strongly by verbal WM demands, with reduced the facilitatory effect for SR pairs but increased the inhibitory effect for WR pairs, compared to a spatial WM task. Critically, high verbal WM load increased the inhibitory effect for WR pairs but did not impact the facilitatory effect for SR pairs. The influence of verbal WM load on responses to WR pairs indicates that the processing of words with a lower degree of semantic relatedness is in particular affected by verbal WM load. In conclusion, the present results showed that working memory type and load influence semantic relatedness judgements but that the direction and size of the impact depend on the strength of semantic relations.

## Data Availability

The dataset generated and analysed during this study is available via the Open Science Framework at https://osf.io/qemk7/; 10.17605/OSF.IO/QEMK7.

## Appendix A

**Table 5:** Lexical characteristics of the 216 word pair triplets: length, word frequency (Zipf values from SUBTLEX-PL; Mandera et al., 2015), and the semantic relatedness values (cosine similarity scores) for the strongly related, weakly related, and unrelated conditions (adapted from Rataj et al., 2023)

Characteristics	Mean	SD (min. - max.)
Target word frequency	3.6	0.4 (2.9 - 4.1)
Target word length	6.3	1.4 (4 - 9)
*Word frequency (prime)*		
strongly related	2.9	0.8 (1.1 - 5.5)
weakly related	3.2	0.8 (1.3 - 5.3)
unrelated	3.5	0.4 (2.9 - 4.7)
*Word length (prime)*		
strongly related	7.2	2.2 (3 - 15)
weakly related	6.4	1.8 (3 - 12)
unrelated	6.7	1.5 (4 - 9)
*Semantic relatedness values*		
strongly related	0.69	.07 (0.48 - 0.86)
weakly related	0.50	.05 (0.42 - 0.75)
unrelated	0.12	.13 (-0.18 - 0.51)

## Footnotes

1 We thank an anonymous reviewer for this suggestion.
